# 4-Azido-2-chloro-6-methyl­quinoline

**DOI:** 10.1107/S1600536809007041

**Published:** 2009-03-06

**Authors:** S. Natarajan, K. Rajesh, V. Vijayakumar, J. Suresh, P. L. Nilantha Lakshman

**Affiliations:** a Department of Physics, Madurai Kamaraj University, Madurai 625 021, India; b Organic Chemistry Division, School of Science and Humanities, VIT University, Vellore 632 014, India; c Department of Physics, The Madura College, Madurai 625 011, India; d Department of Food Science and Technology, Faculty of Agriculture, University of Ruhuna, Mapalana, Kamburupitiya 81100, Sri Lanka

## Abstract

In the title compound, C_10_H_7_ClN_4_, the quinoline ring system is planar [maximum deviation 0.0035 (10) Å]. The crystal structure is stabilized by van der Waals and π–π stacking inter­actions [centroid–centroid distance 3.6456 (17) Å].

## Related literature

For quinoline derivatives as anti-tuberculosis agents, see: Jain *et al.* (2005 ▶).
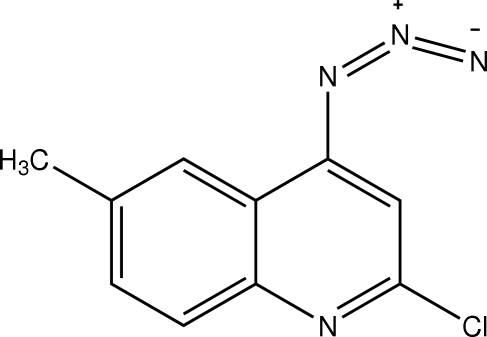


## Experimental

### 

#### Crystal data

C_10_H_7_ClN_4_
*M* *_r_* = 218.65
Triclinic, 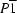

*a* = 6.9517 (4) Å
*b* = 7.6078 (6) Å
*c* = 10.0191 (9) Å
α = 75.694 (7)°
β = 82.147 (8)°
γ = 76.532 (7)°
*V* = 497.57 (7) Å^3^
*Z* = 2
Mo *K*α radiation
μ = 0.35 mm^−1^
*T* = 293 K
0.19 × 0.17 × 0.14 mm

#### Data collection

Nonius MACH-3 diffractometer
Absorption correction: ψ scan (North *et al.*, 1968 ▶) *T*_min_ = 0.935, *T*_max_ = 0.952
2209 measured reflections
1743 independent reflections
1206 reflections with *I* > 2σ(*I*)
*R*_int_ = 0.019
2 standard reflections frequency: 60 min intensity decay: none

#### Refinement

*R*[*F* ^2^ > 2σ(*F* ^2^)] = 0.057
*wR*(*F* ^2^) = 0.180
*S* = 1.08
1743 reflections
137 parameters
H-atom parameters constrained
Δρ_max_ = 0.36 e Å^−3^
Δρ_min_ = −0.35 e Å^−3^

### 

Data collection: *CAD-4 EXPRESS* (Enraf–Nonius, 1994 ▶); cell refinement: *CAD-4 EXPRESS*; data reduction: *XCAD4* (Harms & Wocadlo, 1996 ▶); program(s) used to solve structure: *SHELXS97* (Sheldrick, 2008 ▶); program(s) used to refine structure: *SHELXL97* (Sheldrick, 2008 ▶); molecular graphics: *PLATON* (Spek, 2009 ▶); software used to prepare material for publication: *SHELXL97* .

## Supplementary Material

Crystal structure: contains datablocks global, I. DOI: 10.1107/S1600536809007041/at2729sup1.cif

Structure factors: contains datablocks I. DOI: 10.1107/S1600536809007041/at2729Isup2.hkl

Additional supplementary materials:  crystallographic information; 3D view; checkCIF report

## Figures and Tables

**Fig. 1.**
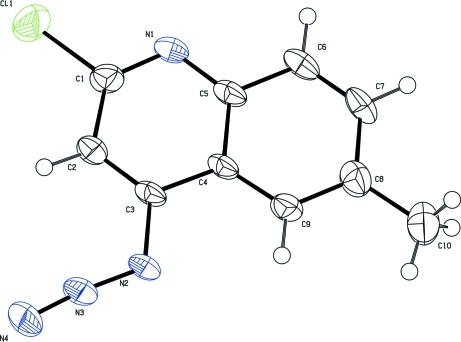
The molecular structure of the title compound, showing 50% probability displacement ellipsoids and the atom-numbering scheme.

## supplementary crystallographic information

### Comment

Quinoline derivatives are a class of important materials as anti-tuberculosis agents (Jain *et al.*, 2005). In the title molecule (Fig. 1), all non-H atoms of the molecule, except atoms Cl1, C10, N2, N3 and N4 are coplanar within 0.0035 (10) Å. Due to 4-azida substitution within the pyridine ring: C2═ C3 bond is longer and the C3—C4 bond is shorter than standard values for C═C (1.334 Å) and C*sp*^2^—C*sp*^2^ (1.455 Å) bond lengths respectively. The dihedral angle between the C3/N2-N4 and C2/C1/N1/C5 rings is 6.16 (11)°.

There is a weak π···π interaction observed between the centres of N1/C1—C5 rings related through the symmetry operator –x, 1-y, 1-z, with centroids separation of 3.6456 (17) Å.

### Experimental

A mixture of 2,4-dichloroquinoline (2.12 g, 10 mmol) and sodium azide (0.650 g, 10 mmol) in DMF (20 ml) was refluxed for 2 h. The progress of the reaction was monitored by TLC. After conforming that the reaction got completed, the reaction mixture was cooled and poured on to the crushed ice with stirring. The solid settled was filtered to dryness and purified over a column of silica gel (60–120 mesh; 50 g) eluting with Petroleum Ether–ethyl acetate (4.5:1.5) to give 4-azido-2-chloro- 6-methylquinoline. The product was re-crystallized from 100% chloroform [mp: 429–430 K, yield: 20%].

### Refinement

The H atoms were placed in calculated positions and allowed to ride on their carrier atoms with C—H = 0.93–0.96 Å and with U_iso_ = 1.2*U*_eq_(C) for CH and *U*_iso_ = 1.5*U*_eq_(C) for CH_3_ groups.

### Crystal data

**Table:**

C_10_H_7_ClN_4_	*Z* = 2
*M**_r_* = 218.65	*F*(000) = 224
Triclinic, *P*1	*D*_x_ = 1.459 Mg m^−^^3^
Hall symbol: -P 1	Mo *K*α radiation, λ = 0.71073 Å
*a* = 6.9517 (4) Å	Cell parameters from 25 reflections
*b* = 7.6078 (6) Å	θ = 2–25°
*c* = 10.0191 (9) Å	µ = 0.35 mm^−^^1^
α = 75.694 (7)°	*T* = 293 K
β = 82.147 (8)°	Block, colourless
γ = 76.532 (7)°	0.19 × 0.17 × 0.14 mm
*V* = 497.57 (7) Å^3^	

### Data collection

**Table:**

Nonius MACH-3 diffractometer	1206 reflections with *I* > 2σ(*I*)
Radiation source: fine-focus sealed tube	*R*_int_ = 0.019
graphite	θ_max_ = 25.0°, θ_min_ = 2.1°
ω–2θ scans	*h* = −1→8
Absorption correction: ψ scan (North *et al.*, 1968)	*k* = −8→9
*T*_min_ = 0.935, *T*_max_ = 0.952	*l* = −11→11
2209 measured reflections	2 standard reflections every 60 min
1743 independent reflections	intensity decay: none

### Refinement

**Table:**

Refinement on *F*^2^	Primary atom site location: structure-invariant direct methods
Least-squares matrix: full	Secondary atom site location: difference Fourier map
*R*[*F*^2^ > 2σ(*F*^2^)] = 0.057	Hydrogen site location: inferred from neighbouring sites
*wR*(*F*^2^) = 0.180	H-atom parameters constrained
*S* = 1.08	*w* = 1/[σ^2^(*F*_o_^2^) + (0.1143*P*)^2^ + 0.0878*P*] where *P* = (*F*_o_^2^ + 2*F*_c_^2^)/3
1743 reflections	(Δ/σ)_max_ < 0.001
137 parameters	Δρ_max_ = 0.36 e Å^−^^3^
0 restraints	Δρ_min_ = −0.35 e Å^−^^3^

### Special details

**Table:**

Geometry. All e.s.d.'s (except the e.s.d. in the dihedral angle between two l.s. planes) are estimated using the full covariance matrix. The cell e.s.d.'s are taken into account individually in the estimation of e.s.d.'s in distances, angles and torsion angles; correlations between e.s.d.'s in cell parameters are only used when they are defined by crystal symmetry. An approximate (isotropic) treatment of cell e.s.d.'s is used for estimating e.s.d.'s involving l.s. planes.
Refinement. Refinement of *F*^2^ against ALL reflections. The weighted *R*-factor *wR* and goodness of fit *S* are based on *F*^2^, conventional *R*-factors *R* are based on *F*, with *F* set to zero for negative *F*^2^. The threshold expression of *F*^2^ > σ(*F*^2^) is used only for calculating *R*-factors(gt) *etc*. and is not relevant to the choice of reflections for refinement. *R*-factors based on *F*^2^ are statistically about twice as large as those based on *F*, and *R*- factors based on ALL data will be even larger.

### Fractional atomic coordinates and isotropic or equivalent isotropic displacement parameters (Å^2^)

**Table:**

	*x*	*y*	*z*	*U*_iso_*/*U*_eq_	
C1	0.1775 (5)	0.7084 (4)	0.2969 (3)	0.0365 (8)	
C2	0.2459 (4)	0.5180 (3)	0.3035 (3)	0.0334 (7)	
H2	0.2656	0.4702	0.2246	0.040*	
C3	0.2820 (4)	0.4060 (3)	0.4303 (3)	0.0294 (7)	
C4	0.2518 (4)	0.4825 (4)	0.5495 (3)	0.0291 (7)	
C5	0.1836 (4)	0.6769 (4)	0.5274 (3)	0.0328 (7)	
C6	0.1508 (5)	0.7579 (4)	0.6436 (3)	0.0432 (8)	
H6	0.1047	0.8853	0.6320	0.052*	
C7	0.1857 (5)	0.6525 (4)	0.7709 (3)	0.0448 (9)	
H7	0.1636	0.7094	0.8454	0.054*	
C8	0.2548 (5)	0.4583 (5)	0.7950 (3)	0.0407 (8)	
C9	0.2849 (4)	0.3774 (4)	0.6839 (3)	0.0354 (7)	
H9	0.3283	0.2495	0.6978	0.043*	
C10	0.2945 (6)	0.3461 (6)	0.9386 (3)	0.0581 (10)	
H10A	0.1764	0.3662	0.9996	0.087*	
H10B	0.3994	0.3836	0.9706	0.087*	
H10C	0.3331	0.2169	0.9371	0.087*	
Cl1	0.13068 (16)	0.85050 (11)	0.13412 (9)	0.0604 (4)	
N1	0.1475 (4)	0.7888 (3)	0.4003 (3)	0.0395 (7)	
N2	0.3501 (4)	0.2108 (3)	0.4540 (3)	0.0396 (7)	
N3	0.3912 (4)	0.1499 (3)	0.3461 (3)	0.0418 (7)	
N4	0.4337 (5)	0.0798 (4)	0.2571 (3)	0.0614 (10)	

### Atomic displacement parameters (Å^2^)

**Table:**

	*U*^11^	*U*^22^	*U*^33^	*U*^12^	*U*^13^	*U*^23^
C1	0.0458 (19)	0.0243 (14)	0.0406 (16)	−0.0080 (13)	−0.0078 (14)	−0.0063 (12)
C2	0.0435 (19)	0.0236 (15)	0.0374 (16)	−0.0062 (13)	−0.0066 (14)	−0.0138 (12)
C3	0.0317 (16)	0.0202 (13)	0.0404 (16)	−0.0029 (12)	−0.0044 (13)	−0.0160 (12)
C4	0.0304 (16)	0.0214 (14)	0.0388 (16)	−0.0021 (11)	−0.0035 (12)	−0.0158 (12)
C5	0.0359 (18)	0.0227 (14)	0.0432 (16)	−0.0032 (12)	−0.0034 (13)	−0.0165 (12)
C6	0.055 (2)	0.0272 (16)	0.053 (2)	−0.0039 (14)	−0.0025 (16)	−0.0249 (14)
C7	0.056 (2)	0.0417 (18)	0.0452 (19)	−0.0095 (16)	0.0017 (16)	−0.0291 (15)
C8	0.044 (2)	0.0449 (18)	0.0385 (17)	−0.0112 (15)	−0.0041 (14)	−0.0171 (14)
C9	0.0400 (19)	0.0255 (14)	0.0426 (17)	−0.0018 (13)	−0.0064 (14)	−0.0139 (13)
C10	0.075 (3)	0.062 (2)	0.041 (2)	−0.013 (2)	−0.0078 (18)	−0.0161 (17)
Cl1	0.0950 (9)	0.0351 (5)	0.0482 (6)	−0.0092 (5)	−0.0201 (5)	−0.0002 (4)
N1	0.0535 (18)	0.0188 (12)	0.0460 (15)	−0.0010 (11)	−0.0058 (12)	−0.0121 (11)
N2	0.0572 (18)	0.0208 (12)	0.0413 (14)	0.0034 (12)	−0.0084 (12)	−0.0164 (11)
N3	0.0582 (19)	0.0204 (12)	0.0487 (16)	−0.0013 (12)	−0.0104 (13)	−0.0146 (12)
N4	0.105 (3)	0.0314 (14)	0.0503 (17)	0.0002 (16)	−0.0140 (17)	−0.0239 (13)

### Geometric parameters (Å, °)

**Table:**

C1—N1	1.298 (4)	C6—H6	0.9300
C1—C2	1.403 (4)	C7—C8	1.413 (4)
C1—Cl1	1.745 (3)	C7—H7	0.9300
C2—C3	1.362 (4)	C8—C9	1.371 (4)
C2—H2	0.9300	C8—C10	1.505 (5)
C3—N2	1.419 (3)	C9—H9	0.9300
C3—C4	1.424 (3)	C10—H10A	0.9600
C4—C9	1.404 (4)	C10—H10B	0.9600
C4—C5	1.415 (4)	C10—H10C	0.9600
C5—N1	1.364 (4)	N2—N3	1.251 (3)
C5—C6	1.416 (4)	N3—N4	1.121 (3)
C6—C7	1.348 (4)		
			
N1—C1—C2	126.1 (3)	C6—C7—C8	122.2 (3)
N1—C1—Cl1	117.0 (2)	C6—C7—H7	118.9
C2—C1—Cl1	116.9 (2)	C8—C7—H7	118.9
C3—C2—C1	117.2 (2)	C9—C8—C7	117.8 (3)
C3—C2—H2	121.4	C9—C8—C10	121.7 (3)
C1—C2—H2	121.4	C7—C8—C10	120.5 (3)
C2—C3—N2	124.0 (2)	C8—C9—C4	121.8 (3)
C2—C3—C4	120.3 (2)	C8—C9—H9	119.1
N2—C3—C4	115.7 (2)	C4—C9—H9	119.1
C9—C4—C5	119.6 (2)	C8—C10—H10A	109.5
C9—C4—C3	124.1 (2)	C8—C10—H10B	109.5
C5—C4—C3	116.3 (3)	H10A—C10—H10B	109.5
N1—C5—C4	123.2 (2)	C8—C10—H10C	109.5
N1—C5—C6	118.8 (2)	H10A—C10—H10C	109.5
C4—C5—C6	117.9 (3)	H10B—C10—H10C	109.5
C7—C6—C5	120.7 (3)	C1—N1—C5	116.7 (2)
C7—C6—H6	119.6	N3—N2—C3	114.1 (2)
C5—C6—H6	119.6	N4—N3—N2	173.6 (3)
			
N1—C1—C2—C3	0.9 (5)	C5—C6—C7—C8	−0.4 (5)
Cl1—C1—C2—C3	−179.7 (2)	C6—C7—C8—C9	−0.5 (5)
C1—C2—C3—N2	179.5 (3)	C6—C7—C8—C10	179.2 (3)
C1—C2—C3—C4	−0.4 (4)	C7—C8—C9—C4	1.0 (5)
C2—C3—C4—C9	179.8 (3)	C10—C8—C9—C4	−178.6 (3)
N2—C3—C4—C9	−0.1 (4)	C5—C4—C9—C8	−0.7 (4)
C2—C3—C4—C5	0.0 (4)	C3—C4—C9—C8	179.5 (3)
N2—C3—C4—C5	−179.9 (3)	C2—C1—N1—C5	−0.9 (5)
C9—C4—C5—N1	−179.9 (3)	Cl1—C1—N1—C5	179.7 (2)
C3—C4—C5—N1	0.0 (4)	C4—C5—N1—C1	0.4 (5)
C9—C4—C5—C6	−0.1 (4)	C6—C5—N1—C1	−179.3 (3)
C3—C4—C5—C6	179.7 (3)	C2—C3—N2—N3	5.8 (4)
N1—C5—C6—C7	−179.6 (3)	C4—C3—N2—N3	−174.3 (3)
C4—C5—C6—C7	0.7 (5)	C3—N2—N3—N4	176 (3)
